# Dietary inflammatory index in relation to the progression of hepatic steatosis and liver fibrosis: evaluation by elastography/Fibroscan

**DOI:** 10.1186/s12876-024-03209-8

**Published:** 2024-04-08

**Authors:** Mahsa Miryan, Sameeah Abdulrahman Rashid, Jamshid Gholizadeh Navashenaq, Davood Soleimani, Mohsen Nematy, Jalal Moludi

**Affiliations:** 1 Student Research Committee, Kermanshah University of Medical Sciences, Kermanshah, Iran; 2 Department of surgery, college of medicine, Hawler Medical University, Kurdistan region, Erbil, Iraq; 3 Noncommunicable Diseases Research Center, Bam University of Medical Sciences, Bam, Iran; 4 Nutritional Sciences Department, School of Nutrition Sciences and Food Technology, Kermanshah University of Medical Sciences, Kermanshah, Iran; 5 Department of Nutrition, Faculty of Medicine, Mashhad University of Medical Sciences, Mashhad, Iran; 6 Research Center of Oils and Fats, Kermanshah University of Medical Sciences, Kermanshah, Iran

**Keywords:** Diet, Nonalcoholic fatty liver disease, NAFLD, Liver fibrosis, Inflammation

## Abstract

One of the proposed mechanisms by which nutrition influences the progression of hepatic steatosis to fibrosis is inflammation. The study investigated how the inflammatory potential of the diet affects the risk of liver damage in patients with nonalcoholic fatty liver disease (NAFLD), a condition where fat accumulates in the liver. This cross-sectional study included 170 outpatients with newly diagnosed NAFLD. This study used a device called Fibroscan® to measure the degree of liver fibrosis, which is the scarring of the liver tissue due to chronic inflammation. The study also used a tool called the Dietary Inflammatory Index (DII) to measure the inflammatory potential of the diet based on the intake of different foods and nutrients. In the findings of the study, patients with more severe fat accumulation in the liver (hepatic steatosis) had higher DII scores, meaning they had more inflammatory diets. The study also found that higher DII scores were associated with higher weight and body mass index (BMI). One standard deviation (SD) increase in DII scores was associated with a 0.29 kilopascal (95% CI: 0.10–0.44; P-value 0.001) increase in the mean liver stiffness, an indicator of liver fibrosis. The study concluded that patients with higher DII scores had a higher risk of developing liver fibrosis than those with lower DII scores, even after adjusting for confounding factors (odds ratio: 5.89; P-value: 0.001). The study suggested that eating less inflammatory foods may help prevent or slow down the progression of hepatic steatosis and liver in patients with NAFLD.

## Introduction

Non-alcoholic fatty liver disease (NAFLD) is becoming the leading cause of chronic liver disease in the world. This disease currently affects about a quarter of adults worldwide [1]. NAFLD includes a wide spectrum of diseases ranging from simple hepatic steatosis to steatohepatitis that can progress to liver fibrosis and hepatocellular carcinoma. A meta-analysis of paired-biopsy studies has estimated the annual fibrosis progression rate of 0.07 and 0.14 stages in patients with simple hepatic steatosis and steatohepatitis, respectively [2]. Currently, there are no specific FDA-approved pharmaceutical agents for the treatment of NAFLD.

The underlying cause of NAFLD is unclear but appears to be multifactorial and results from the interaction of genetic and environmental factors. Inflammation is considered an important molecular mechanism in the progression of hepatic steatosis and liver fibrosis [3]. Also, diet is one of the main environmental factors that can modulate molecular mechanisms underlying inflammation. Previous studies have shown that some individual nutrients and foods have anti-inflammatory potentials (e.g., omega-3 fatty acids, zinc, vitamin D, vitamin E, nuts, fish, and berries) or pro-inflammatory potentials (e.g., saturated and trans-fats, proceed meats, and refined grains) [4, 5]. Such analyses in nutritional studies ignore various aspects of the diet such as the cumulative effects of different nutrients and foods eaten together [6].

Recently, there has been growing interest in using dietary indices and patterns to predict the overall diet effects on the risk of non-communicable diseases and mortality. These approaches in nutritional epidemiology overcome these limitations and are also more translatable in terms of public health messaging. In this context, the dietary inflammatory index (DII) is designed to quantify the overall inflammatory potential of diet [7]. DII has been established to predict the levels of pro-inflammatory and anti-inflammatory markers in humans and the risk of inflammation-related diseases such as cardiovascular diseases [8], rheumatoid arthritis [9], metabolic syndrome [10], and diabetes mellitus [11]. Knowledge concerning the role of overall diet inflammatory potential in the progression of hepatic steatosis and liver fibrosis can help elucidate therapeutic plans for patients with NAFLD. Therefore, our study aimed to evaluate whether adherence to diets with lower inflammatory potential, indicated by lower DII scores, may help prevent or slow down the progression of hepatic steatosis and liver fibrosis in patients with NAFLD.

## Materials and methods

### Study design and participants

The cross-sectional study was conducted on data from adult patients who were referred to the gastrointestinal clinic of Imam Reza Hospital in Mashhad, Iran. Three hundred consecutive outpatients who had newly diagnosed NAFLD by radiologic imaging techniques were enrolled and then assessed for eligibility criteria. The inclusion criteria were an age of 20 to 60 years, radiologic evidence of hepatic steatosis, the absence of secondary causes of hepatic steatosis (e.g., hepatitis B and C virus, autoimmune diseases, steatogenic medications), and written informed consent. The exclusion criteria were a history of daily alcohol consumption above 20 g in women and 30 g in men, consistent use of dietary antioxidant supplements such as vitamin E, and change in dietary habits since NAFLD diagnosis. After screening the participants, one hundred and eighty-nine patients who fulfilled the eligibility criteria underwent further radiological examination (Fibroscan®) and dietary assessment. The study was accepted by the Ethics Committee of Mashhad University of Medical Sciences (IR.MUMS.MEDICAL.REC.1397.121) and then was accepted by the Ethics Committee of Kermanshah University of Medical Sciences (IR.KUMS.REC.1400.789). This study was conducted in accordance with the Declaration of Helsinki.

### Non-invasive radiological liver assessment

An expert radiologist performed liver examinations using a single Fibroscan® machine (Echosens, Paris, France) with probe M. Fibroscan® is a one-dimensional transient elastography system that provides a novel non-invasive technique to predict liver fibrosis and steatosis. This system utilizes the shear wave with mild amplitude and low frequency to measure the liver stiffness measurement (LSM) and controlled attenuation parameter (CAP). Fibroscan® is operator-independent and reproducible with excellent inter-observer agreement and intra-observer agreement for predicting hepatic steatosis and liver fibrosis [12].

This procedure was performed for all eligible patients in the fasting state and through the intercostal space. Reliable Fibroscan® procedures had at least 10 valid measurements with a success rate of more than 60% and an interquartile range/median of less than 30% [13]. The median value of successful LSM > 5.3 kilopascals (kPa) was set to diagnose the presence of hepatic fibrosis in patients with NAFLD, based on METAVIR criteria with the area under the receiver operating characteristic curve (AUROC) of 0.879 [14]. The optimal cut-off points of CAP value for steatosis grade Ι (11∼33%), steatosis grade ΙΙ (34∼66%), and steatosis grade ΙΙΙ (67∼100%) have been determined 238 (AUROC = 0.91), 259 (AUROC = 0.95), and 292 (AUROC = 0.89) dB/m, respectively [15]. Patients who had a CAP value < 238 or unreliable LSM in elastography examination were excluded from the data analysis.

### Dietary inflammatory index

The Dietary Inflammatory Index (DII) is a dietary assessment tool that is based on a compilation of scientific literature regarding the effects of various nutrients and foods on the levels of pro-inflammatory and anti-inflammatory mediators. These mediators include molecules such as tumor necrosis factor-Alpha (TNF-α), interleukin-1Beta (IL-1β), IL-4, IL-6, IL-10, and C-reactive protein (CRP) [7]. Briefly, in the scoring algorithm of DII, dietary components with the potential to suppress inflammation had an inflammatory effect score from − 1 (maximally anti-inflammatory) to 0. In our study, these anti-inflammatory nutrients and foods includes fiber, vitamin A, vitamin C, vitamin D, vitamin E, vitamin B1, vitamin B2, vitamin B3, vitamin B6, vitamin B9, zinc, selenium, magnesium, polyunsaturated fatty acids, monounsaturated fatty acids, ω − 3 fatty acids, ω − 6 fatty acids, β-carotene, garlic, ginger, onion, saffron, turmeric, pepper, thyme/oregano, rosemary, green/black tea, alcohol, and caffeine. Dietary components that have the potential to increase inflammation are scored from + 1 (maximally inflammatory) to 0, including energy, protein, carbohydrates, total fat, saturated fatty acids, trans fat, cholesterol, vitamin B12, and iron. Dietary intakes were assessed by three 3-day food records from each patient. The amount of each food item was converted to weight (grams/day) using the Iranian household measures. These values were then transformed to energy and nutrients using Nutritionist IV software (N-Squared Computing, Salem, OR, USA). Patients who had a reported energy intake of < 800 kcal/day (under-reporting) or > 4200 kcal/day (over-reporting) were excluded from the data analysis.

While calculating the DII, we divided the intake of each dietary parameter by the applicable global mean intake to get a Z score, which is equal to (individual mean intake - global mean intake)/global standard deviation. This Z score was translated to a percentile score, which was then multiplied by two and deducted from “+1”. The “food parameter-specific DII score” is created by multiplying this number by the appropriate inflammatory impact score. The total DII score of each person’s diet is then calculated by adding all of the DII values for each dietary parameter [7].

### Biochemical assessment

After a 10-hour fast, blood samples from each patient were taken. The serum was then spun at 3000 rpm for 15 min at 4° C to separate it from the coagulated blood. Fasting blood sugar, total cholesterol, triglycerides, high-sensitivity C-reactive protein (hs-CRP), and gamma-glutamyl transferase (GGT) concentrations were determined enzymatically on a biochemistry autoanalyzer (Alfa-Classic; Tajhizat Sanjesh Co., Ltd., Iran) using commercial kits (Pars Azmoon, Tehran, Iran).

### Statistical analysis

All statistical analyses were done on SPSS software version 16 (SPSS Inc., Chicago, USA). The minimum sample size was estimated at 97 patients based on a 55.3% prevalence of hepatic fibrosis among NAFLD patients with a precision of 0.1 at a two-tailed significance level of 0.05 [16, 17]. We raised the necessary sample size to account for participant dropout and erroneous or incomplete Fibroscan® data. With the use of the 2 test, the distribution of qualitative characteristics across the tertiles of DII scores was compared. Using the Kolmogorov-Simonov test, we examined the normality of the distribution of quantitative data. With the use of the one-way ANOVA test and the following Bonferroni Post hoc test, normally distributed variables were compared among the tertiles of DII scores, and non-normally distributed variables were compared using the Kruskal-Wallis test. Binary logistic regression was used to calculate the odds ratio (95% confidence interval) of hepatic fibrosis across the tertiles of DII scores in both the unadjusted and adjusted models. To identify changes in variables for each SD rise in the DII score of diet, we also used univariate and multivariate linear regression models. Statistics were deemed significant when P values were less than 0.05.

## Results

Three hundred participants were screened for this study. Two hundred and twenty-two of them met the inclusion criteria, and 189 of them underwent transient elastography (Fibroscan®) testing and returned the completed food record questionnaires (response rate: 85.1%). Nineteen subjects also were eliminated from the data analysis because of implausible calorie intake (N: 9), inconsistent LMS values (N: 6), and CAP scores below 238 dB/m (N:4). The final analysis included 170 individuals, of whom 61 subjects had grade I hepatic steatosis (35.9%), 66 subjects had grade II hepatic steatosis (38.8%), and 43 subjects had grade III hepatic steatosis (25.3%). Also, 94 of these individuals had hepatic fibrosis. The majority of participants were female (63.5%) with a mean age (± SD) of 39.94 (± 12.98) years.

According to the tertiles of DII scores, Table 1 displays the participants’ demographic information. In terms of cardiovascular diseases, diabetes mellitus, smoking, educational attainment, and use of nutritional supplements and anti-diabetic medications, the distribution of individuals across the DII tertiles did not vary substantially (P-value > 0.05). Individuals in the highest tertile of the DII scores had higher weight and BMI than those in the lowest tertile of the DII scores. In comparison to subjects in the lowest tertile of the DII scores, individuals in the highest tertile were more likely to have greater weights (88.47 ± 16.29 vs. 78.56 ± 15.34 Kg; P-value: 0.002) and BMIs (30.95 ± 4.18 vs. 30.95 ± 4.18 Kg/m^2^; P-value: 0.001). Moreover, there was a significant linear trend in weight across DII score tertiles (P-trend: 0.001) and BMI across tertiles (P-trend: 0.001). Each 1 SD increase in DII score was linked to average weight and BMI increases of 3.37 kg (95% CI: 0.98–5.77; P-value: 0.006) and 1.06 Kg/m^2^, respectively. Those with greater tertiles of DII scores showed higher levels of hepatic steatosis (P-value: 0.037). The levels of hepatic steatosis and DII scores were significantly correlated (Spearman correlation coefficient: 0.215; P-value: 0.005).

**Table 1:** Demographic characteristics of the study participants across tertiles (T) of DII scores

Variables		Tertiles of Dietary Inflammatory Index			P-value
		T_1_ *Low inflammatory* *diet*	T_2_	T_3_ *High inflammatory* *diet*	
Number		58	56	56	-
DII score		-1.55–1.35	1.40–2.40	2.40–3.68	-
Male, n%		24 (41.4%)	17 (30.4%)	21 (37.5%)	0.465†
Age, years		39.24 ± 13.06	40.82 ± 13.90	39.77 ± 12.10	0.806 ‡
Weight, kg		78.56 ± 15.34	85.17 ± 15.13	88.47 ± 16.29	0.003‡
BMI, kg/m²		27.73 ± 3.77	29.92 ± 4.7	30.95 ± 4.18	0.001‡
Physical activity, MET-h/week		9.43 ± 5.9	10.6 ± 5.2	9.85 ± 4.8	0.941‡
CVD, n%		12 (20.7%)	12 (21.4%)	5 (8.9%)	0.141†
Diabetes mellitus, n%		8 (13.8%)	10 (17.9%)	8 (14.3%)	0.807†
Current Smoker, n%		8 (13.8%)	13 (23.2%)	11 (19.6%)	0.429†
Hepatic steatosis, n%	Mild	27 (46.6%)	23 (41.1%)	11 (19.6%)	0.037†
	Moderate	20 (34.5%)	19 (33.9%)	27 (48.2%)	
	Severe	11 (19%)	14 (25%)	18 (32.1%)	
University education, n%		10 (17.2%)	6 (10.7%)	7 (12.5%)	0.843†
Anti-diabetic drugs use, n%		14 (24.1%)	13 (23.2%)	12 (21.4%)	0.941†
Dietary supplement use, n%		7 (12.3%)	8 (14.3%)	6 (10.7%)	0.848†

*Abbreviations* BMI, body mass index; CVD, cardiovascular disease, MET-h, metabolic equivalent hours

*Notes* Data are reported as mean and standard deviation or percentage (Frequency) as appropriate

† P-values were calculated using the chi-squared test

‡ P-values were calculated using the one-way ANOVA test

Table 2 displays participant biochemical data according to the tertiles of DII scores. In terms of TG, total cholesterol, FBS, ALT, AST, and GGT, the tertiles of the DII scores were not different. There was no significant correlation between these metabolic markers and DII scores, according to linear regression analysis. In both crude and energy-adjusted models, there was no discernible linear trend in the liver stiffness measurement (LSM) across the tertiles of the DII scores (P-trend > 0.05), as shown in Table 3. A significant linear trend in LSM was seen throughout the tertiles of the DII scores after further adjustment for age, sex, BMI, physical activity, smoking, and education status (Model II) (P-trend: 0.003). The LSM value was greater for subjects in the highest tertile of DII scores than for those in the lowest tertile (6.08 ± 0.68 vs. 5.69 ± 0.60; P-value: 0.003). When accounting for the presence of hepatic steatosis, this tendency became more pronounced (P-value: 0.001). Additional investigation revealed that for each 1 SD increase in the DII scores, inflammatory diets were independently linked with a rise in LSM of around 0.29 kPa (95% CI: 0.1–0.44; P-value: 0.001).

**Table 2:** Biochemical parameters of study participants across the tertiles of dietary inflammatory index

Variables	Tertiles of Dietary Inflammatory Index				Beta Coefficient (95% CI) per SD
	T_1_ *Low inflammatory* *diet*	T_2_	T_3_ *High inflammatory* *diet*	P-trend	
DII	-1.55–1.35	1.40–2.40	2.40–3.68	-	-
TG, mg/dL	144.45 ± 78.36	116.82 ± 61.64	131.13 ± 61.53	0.322	-10.98 (-21.54–0.419)
TC, mg/dL	169.61 ± 44.72	172.07 ± 33.51	185.29 ± 39.74	0.057	4.05 (-2.39–10.49)
FBS, mg/dL	106.34 ± 32.77	97.87 ± 19.51	102.92 ± 23.22	0.515	1.04 (-3.12–5.21)
ALT, IU/dL	26.89 ± 18.86	26.26 ± 16.76	31.04 ± 15.92	0.250	0.57 (-2.22–3.36)
AST, IU/dL	23.81 ± 11.7	22.25 ± 9.67	26.23 ± 10.35	0.272	0.49 (-1.25–2.24)
GGT, IU/dL	28.62 ± 12.07	28.95 ± 14.62	27.92 ± 8.96	0.795	-0.34 (-2.52–1.84)

*Abbreviations* TG: Triglyceride, TC: total cholesterol; FBS: Fasting blood sugar, ALT: alanine aminotransferase; AST: aspartate aminotransferase; GGT: gamma-glutamyl transferase

*Notes* Values are shown mean ± standard deviation or mean (95% confidence interval)

P-trends were calculated using the one-way ANOVA test

Beta coefficients (95%CI) were obtained using the linear regression test

**Table 3:** Association between the liver stiffness measurement (LSM) and the tertiles of dietary inflammatory index scores

Models	Tertiles of Dietary Inflammatory Index				Beta Coefficient (95% CI) per SD
	T_1_ *Low inflammatory* *diet*	T_2_	T_3_ *High inflammatory* *diet*	P-trend	
LSM in crude model; kPa	5.46 ± 2.03	6.06 ± 3.70	6.22 ± 1.57	0.123	0.043 (-0.016–0.101)
LSM in adjusted model I; kPa	5.90 ± 0.12	5.91 ± 0.06	5.90 ± 0.07	0.797	0.232 (-1.493–1.956)
LSM in adjusted model II; kPa	5.69 ± 0.60	5.93 ± 0.74	6.08 ± 0.68	0.003	0.264 (0.048–0.480)
LSM in adjusted model III; kPa	5.63 ± 0.73	5.91 ± 0.88	6.17 ± 0.71	0.001	0.287 (0.102–0.437)

*Abbreviations* kPa: kilopascals

Values are shown mean ± standard deviation or mean (95% confidence interval)

*Model* I. Adjusted for energy intake

*Model* II. Further adjusted for age, sex, BMI, physical activity, smoking, and education status

*Model* III. Further adjusted for hepatic steatosis status

P-trends were obtained using the one-way ANOVA test

Beta coefficients (95%CI) were obtained using the linear regression test

**Fig. 1:**
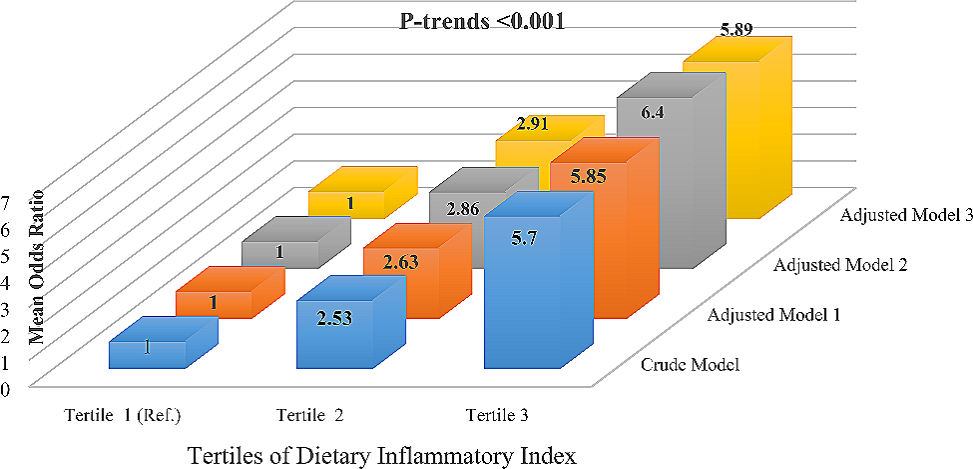
Odds ratio of having hepatic fibrosis across the tertiles of dietary inflammatory index score. *Notes* Odds ratios were obtained using the binary logistic regression test. *Model (1)* Adjusted for energy intake. *Model (2)* Further adjusted for age, sex, BMI, physical activity, smoking, and education status. *Model (3)* Further adjusted for hepatic steatosis status

The odds ratio (OR) of hepatic fibrosis for each tertile of DII scores is shown in Fig. 1. According to the crude model, subjects in the highest tertile of DII scores had a greater likelihood of developing hepatic fibrosis than those in the lowest tertile of DII scores (OR: 5.70; 95% CI: 2.53–12.84; P-trend 0.001). This association remained significant after the adjustment of potential confounders such as energy (Model 1), age, sex, BMI, physical activity, smoking, education status (Model 2), and degrees of hepatic steatosis (Model 3). The probabilities of hepatic fibrosis showed a statistically significant rising trend in all models (P-trend 0.001). Additional investigation revealed that for each SD increase in the DII score of the diet, inflammatory diets were independently linked with around 87% greater chances of developing hepatic fibrosis in individuals with NAFLD (OR: 1.87; 95%CI: 1.28–2.72; P-value: 0.001).

## Discussion

The main finding of the current study is a direct relationship between inflammatory potential of diet, as measured by the DII score, and the development of hepatic steatosis and liver fibrosis in individuals with NAFLD. This finding was independent of the potential risk factors for the development of hepatic fibrosis, including hepatic steatosis levels, sex, and BMI. To our knowledge, there are no published studies documenting the role of pro-inflammatory diets on the severity of hepatic steatosis along with the development of liver fibrosis in NAFLD patients.

Our study showed a strong correlation between DII scores and hepatic steatosis levels in patients with NAFLD, a finding that is consistent with previous studies. Soltanieh et al. showed that adherence to pro-inflammatory diets (greater DII score) was significantly associated with the increased risk for the presence of NAFLD in subjects with type 2 diabetes mellitus [18]. Similarly, in a cross-sectional analysis of data in the Fasa Cohort Study, Valibeygi et al. showed that the DII scores were associated with the increased risk for the presence of NAFLD, as measured by fatty liver index [19]. In a case-control study on newly diagnosed NAFLD cases, Farhadnejad et al. showed that the inflammatory potential of diet measured by the DII score and empirical dietary inflammatory patterns (EDIP) was significantly associated with the odds for the presence of NAFLD in ultrasonography scan [20]. In another case-control study, adherence to diets with high DII scores was significantly associated with the increased risk odds for the presence of NAFLD [21]. Cantero et al. showed that the DII scores were positively associated with fatty liver index and serum ALT, AST, and GGT concentrations [22]. Ramírez-Vélez et al. showed that adherence to pro-inflammatory diets was significantly associated with the increased fatty liver index, AST to ALT ratio, and serum ALT and GGT concentrations in the US adult population [23]. However, they found no relationship between the DII scores and liver damage parameters measured by transient elastography such as LSM and CAP. In the Amol Cohort Study, Doustmohammadian et al. revealed that the DII scores significantly increased the risk for the presence of NAFLD as measured by ultrasonography in men and women. They revealed that the DII score was not associated with the risk for the presence of NAFLD as measured by fatty liver index in women. By contrast, there were no significant associations between the DII scores and the risk for the presence of NAFLD as measured by hepatic steatosis index in men [24]. The inconsistency of the results between some mentioned studies is probably due to using different methods in the evaluation of NAFLD in epidemiological studies. Taken together, a recent meta-analysis of 10 studies comprising 242,006 participants showed that higher adherence to pro-inflammatory diets significantly increased the risk of NAFLD [25]. It seems that the relationship between inflammation and hepatic steatosis is profoundly influenced by insulin resistance [26, 27]. In fact, a chronic inflammatory state causes insulin resistance and, consequently, hyperinsulinemia, which upregulates lipogenic transcription factors to cause fatty acid production in the liver and white adipose tissue [28]. Hence, one of the mechanisms by which nutrition may influence the progression of hepatic steatosis is inflammation.

Our study also showed an independent relationship between DII scores and the development of liver fibrosis in NAFLD patients. Few studies have investigated the relationship between the inflammatory potential of diet and the risk of liver fibrosis in NAFLD patients. A cross-sectional data consisting of 10,052 participants in the National Health and Nutrition Examination Survey (NHANES) from 2005 to 2016 showed that the DII scores were significantly associated with odds of liver fibrosis measured by NAFLD fibrosis score (NFS) in men but not in women [29]. In another cross-sectional data consisting of 5,506 participants in the NHANES from 2005 to 2016 showed that adherence to diets with high DII scores was significantly associated with the increased risk of liver fibrosis measured by NFS [30]. Previous investigations revealed adherence to Mediterranean diets is linked to a lower risk of the liver fibrosis in patients with NAFLD [31]. The Mediterranean diets are rich in nutrients and foods with anti-inflammatory potential, such as white meats, vegetables, vegetable oils, fresh fruits, nuts, and low-fat dairy products [32, 33]. Similarly, following a healthy diet that focuses on eating lots of anti-inflammatory foods like low-fat dairy, white meat, nuts, vegetables, fruits, tea, and coffee is linked to a lower risk of liver fibrosis, as opposed to following a Western diet that prioritizes eating lots of inflammatory foods like red meat, refined grains, potatoes, eggs, soft drinks, and hydrogenated fats [17]. It has been shown that diets with high DII scores may raise the levels of inflammatory mediators including IL-6, IL-1, and TNF-α. Hepatic stellate is stimulated by inflammatory mediators to become active, proliferate, migrate, and transdifferentiate into myofibroblast-like cells in the damaged areas of the liver. This plays a crucial role in the transition of hepatic steatosis to fibrosis [34].

Hence, decreasing inflammation via diet might be one of the key treatment methods to avoid the development of hepatic steatosis to fibrosis in individuals with NAFLD.

There were several limitations and strengths to this study as follows. First, the cross-sectional design of the present research makes it impossible to infer causality. Therefore, longitudinal and clinical trial studies are required to evaluate the effect of the DII scores on liver status. Second, dietary intakes were assessed by three 3-day food records. This tool has been proposed as a valuable method for evaluation of actual intakes and reduction of recall errors, although it may limit the ability to accurately describe usual intakes. Third, in the absence of liver biopsy as the gold standard method to diagnose hepatic steatosis and fibrosis, we used fibroscan which is a non-invasive technique and provides patients with NAFLD great diagnostic accuracy and outstanding repeatability for hepatic steatosis and fibrosis compared to non-invasive diagnosis indexes (i.e., the FLI and NFS). The high response rate for food records (85.1%), homogeneity of the study population, accounting for potential confounding factors such as physical activity, evaluation of actual intakes, and eradication of memory bias by the use of food records are further benefits of our research. Further studies with longitudinal or clinical trial design and large sample size needed to clarify the causal relationship and to confirm these findings.

## Conclusion

This study showed that the inflammatory potential of diets is associated with liver damage. The consumption of a pro-inflammatory dietary pattern might contribute to the development of hepatic steatosis and liver fibrosis in individuals with NAFLD. This study suggests that a well-designed precision diet including putative anti-inflammatory components could specifically prevent and ameliorate the development of NAFLD in addition to obesity. Further studies with longitudinal or clinical trial design and large sample size are needed to clarify the causal relationship and to confirm these findings.

## Data Availability

The data that support the findings of this study are available on request from the corresponding author. The data are not publicly available due to privacy or ethical restrictions.
